# Editorial: Immersive media in connected health—volume II

**DOI:** 10.3389/fdgth.2024.1425769

**Published:** 2024-05-20

**Authors:** P. E. Antoniou, D. Economou, A. Athanasiou, G. Tsoulfas

**Affiliations:** ^1^ Lab of Medical Physics and Digital Innovations, Department of Medicine, School of Health Sciences, Aristotle University of Thessaloniki, Thessaloniki, Greece; ^2^ School of Computer Science, University of Westminster, London, United Kingdom; ^3^ Department of Transplantation Surgery, Ippokrateio General Hospital/Aristotle University of Thessaloniki, Thessaloniki, Greece

**Keywords:** extended reality, medical education, rehabilitation, assistive robotics, co-creation, ADHD, neurosurgery

## Abstract

Immersive media, particularly Extended Reality (XR), is at the forefront of revolutionizing the healthcare industry. Healthcare provides XR with “silver bullet” use cases that add value and societal effect to the technology. Healthcare interventions frequently require imaging or visualization to be applied correctly, and the sensation of presence that XR can provide is crucial as a training aid for healthcare learners. From anatomy to surgical training, multimodal immersion in the reality of a medical situation increases the impact of an XR resource compared to the usual approach. Thus, healthcare has become a specialized focus for the immersive media sector, with a multitude of development and research underway. This research subject, which followed on from the previous one, yielded an eclectic group of works spanning the gamut of immersive media applications in healthcare. The underlying theme in these works remains a consistent focus on calibrating, validating, verifying, and standardizing procedures, instruments, and technologies in order to constantly rigorously streamline the means and materials that will integrate immersive technologies in healthcare. In that spirit, we share the findings from this research topic as a motivator for rigorous and evidence-based use of immersive media in digital and connected health.

**Editorial on the Research Topic** Immersive media in connected health—volume II

## Introduction

This research topic is the follow through to a previous one that opened with the statement: “According to fortune business insights the market size of eXtended Reality (XR) […] is estimated to grow to 30.4 bil. USD by 2027” (1). Three years and a pandemic later, in 2023, the global XR market was valued at 131.54 bil. USD with projected combined aggregate growth rate (CAGR) of 32% per annum (2). Academically, a Google scholar search with the key words “Healthcare” and “extended reality” provided 8,550 hits with more than half of them (4,590) in 2023 and 2024 on the 22/04/2024. The next day (23/04/2024) the same search produced 10 more hits for a total 8,560 hits (4,600 between 2023 and 2024). These simple metrics demonstrate that immersive media, specifically virtual, augmented and mixed reality (comprising the eXtended Reality—XR—spectrum) are in the forefront of disrupting the healthcare sector.

It would be easy to dismiss this boom as the peak of the inflated expectations in the Gartner hype cycle (3), however, unlike other disruptors (e.g., generative AI), XR has been around for quite some time. While the preliminary technology attempts of the 1980s are now only historically and philosophically identified as VR technologies (4), they did serve, with their commercial failure, as a kind of inoculation against the initial hype spike. In fact, healthcare has become a bespoke target for the immersive media industry, exactly as a response to the wild promises of ubiquitous “virtual citizenship” that still does not find widespread reach (5).

Healthcare offers to immersive media (especially XR) the “silver bullet”, use cases that provide value and impact to this technology. Almost all healthcare interventions require some kind of imaging reference to appropriately apply. The capacity for seeing, together with the patient, the actual medical images, e.g., overlayed on a surgical field, is an invaluable aid in the operating theatre. Furthermore, the visual impact and sense of presence that XR can offer is invaluable as an educational aid for the healthcare learner. From anatomy to surgical training, the multisensory immersion in the reality of a medical case multiplies the educational impact of an XR resource against the standard study material (6, 7).

In that environment, this research topic, following through on the previous one, garnered an eclectic collection of works spanning the spectrum of immersive media use cases in healthcare.

## Contributions' outline

Immersivity of available clinical neuroimaging during neurological surgery presents as a key issue in real-time surgical guidance and is incorporated through directions such as neuronavigation, live AR enhancement and integration (8). Surgical immersion through these efforts suffers from real time deformations of the operative field as the operation progresses. Therefore the need for quick, robust and reliable reacquisition of neuroimaging during surgery should be eventually addressed (9), especially as more intricate immersive media become available for intraoperative use (10). Chrisochoides et al. perform a comparative review of physics-based deformable registration methods for image-guided neurosurgery and find that currently a combination of approximation-based and geometry-based point and element outlier rejection improves the rigid registration error margins and approximate time constraints for real operative scenarios. The authors raise questions and open problems for the robust estimation and improvement of registration error due to sparse, noisy, and incomplete data.

Moving on to the field of collaborative and assistive robotics, which, along with various converging technologies, have seen rapid developments enhancing their usability and clinical applicability (11), while implementations in XR environments and gamification (12) expand their potential through immersiveness, empowerment and improved overall user experience. Although the role of emotions and affective states are considered crucial to the learning process, their implications on rehabilitation process have not been sufficiently studied (13). Rodrigues et al. investigate usability and user experience of a system integrating augmented reality technology to a collaborative robot for rehabilitation, aiming at studying factors like motivation and adherence to the treatment, the quality of the movement achieved and repeatability. Their analyses showed that the cohort of users found the system easy and enjoyable while expert opinion algo gave a positive outlook.

While studying user experience and affective analytics can help steer XR development through the iterative process (14), on the other hand, incorporating an *a priori* introduction of co-creation methodologies for immersive content such as Virtual, Augmented or Mixed Reality (VR/AR/MR) resources is considered relevant not only in the development of rehabilitation and applied healthcare (15) but also in healthcare education (6). Antoniou et al. in their work study the formulation of evidence-based, optimized workflows that streamline the process of creating immersive content and allow for rapid expansion of innovative educational approaches in healthcare curricula. They study the views of healthcare educators and healthcare education developers after participation in a series of co-creation sessions. Their aim, based on the insight received, was to determine best practice for design and development of XR educational resources.

Immersive media interventions for attention deficit disorders are prolific given XR's attention enhancing experience (16). Goh et al., in their work published in this topic, employed a VR classroom to validate a continuous performance cognitive attention test on a large sample of neurotypical male and female children between the ages of 6 and 13. The dataset collected also produced an evidence based progression of neurotypical attention performance through developmental years highlighting gender-specific performance variances. The database produced is also a valid benchmark that can be used as comparison with children being assessed for ADHD or other neurocognitive conditions.

## Concluding remarks

From this brief overview, It becomes clear that, while the subjects diverge, the overarching theme in these works retains a consistent direction towards calibration, validation, verification and standardisation of methods, instruments and technologies. In short, this research topic focuses not so much on “just another pilot study” but on the constant endeavours for rigorous streamlining of the means and materials that will integrate immersive technologies in healthcare. In that spirit we invite the reader to explore and hopefully utilise this research to their future endeavours for immersive media in digital and connected health.
